# Efficacy and safety of low-intensity extracorporeal shock wave therapy versus on-demand tadalafil for erectile dysfunction

**DOI:** 10.1080/2090598X.2022.2090134

**Published:** 2022-06-24

**Authors:** Fouad Zanaty, Atef Badawy, Hossam Kotb, Fatma Elsarfy, Baher Salman

**Affiliations:** Urology Department, Menoufia University Hospitals, Faculty of Medicine, Shibin Elkom, Egypt

**Keywords:** Erectile dysfunction, tadalafil, low intensity extracorporeal shock wave therapy, LIESWT

## Abstract

**Objective:**

To compare outcomes of low-intensity extracorporeal shock wave therapy (LIESWT) versus 20 mg of Tadalafil in Erectile dysfunction (ED) patients.

**Materials and Methods:**

We performed a prospective study of 51 men with ED. Twenty-five were in the LIESWT group and 26 in the Tadalafil group. Patients in the LIESWT group received 6 sessions (2 per week) with an average of 6,000 shocks per session with the PiezoWave^2^ unit. Other patients self-administered Tadalafil on demand. The outcomes were assessed using the International Index of Erectile Function (IIEF-5) score, Erection Hardness Score (EHS) and Self-Esteem And Relationship (SEAR) questionnaire before, at 6 and 12 weeks after treatment. Treatment-related side effects and costs were recorded too.

**Results:**

The mean age in the LIESWT group was 43.7 years old, and in the Tadalafil group was 47 years old. After the 6 and 12-week follow-ups, both groups showed significant improvement when comparing the baseline values to the follow-up variables for all IIEF-5, EHS, and SEAR (P < 0.05). There was a notable statistical difference between the two groups regarding the side effects, as the shockwave group was with mild side effects (8%), while the Tadalafil group (44%) of patients had side effects (p < 0.05). This cost difference is statistically significant (p < 0.001). LIESWT is more costly compared to Tadalafil.

**Conclusion:**

LIESWT has a comparable short-term therapeutic efficacy with higher safety outcomes than on-demand 20 mg of Tadalafil for ED patients.

## Introduction

Erectile dysfunction (ED) is a universal male sexual disorder impacting about 52% of men between 40–70 years old and influencing couples’ mental health, self-esteem, and satisfaction with sexual intercourse [1]. Phosphodiesterase type 5 inhibitors (PDE5Is) (Sildenafil, Avanafil, Vardenafil, and Tadalafil) are the first-line therapy for ED management by relaxing penile smooth muscle cells with both corpora cavernosal filling [2,3]. Ten or 20mg of an oral Tadalafil tablet is administered in on-demand doses according to the patient’s adverse event and response [4]. Some ED patients dismally responded to PDE5Is, so they need to start local treatments with topical/intraurethral alprostadil, vacuum erection devices, intracavernosal injection, or interfere with surgical implantation with penile prostheses [5]. Therefore, a non-invasive treatment may improve erectile function as low-intensity extracorporeal shock wave therapy (LIESWT) is desired.

LIESWT has been used in many vasculogenic diseases with peripheral artery illness, such as vasculogenic ED. This is due to the release of angiogenic factors and recruitment of circulating endothelial progenitor cells based on the associated cell membrane microtrauma and mechanical stress [6]. Currently, Tadalafil has been validated as a treatment for ED. LIESWT has gained considerable popularity for the treatment of ED because it provides a curative and rehabilitative effect for ED [7,8] So this study aims to compare the outcomes of LIESWT versus oral on-demand 20 mg of Tadalafil medical treatment in the management of ED patients.

## Methods

Between July 2021 and January 2022, we performed a prospective study of 51 men with ED. Twenty-five patients were in the LIESWT group, and the other 26 patients were on oral Tadalafil treatment. All patients were adults with ED for at least 12 months and in a stable marriage relationship. Our study has received ethics approval from our university committee under the Declaration of Helsinki. Written informed consent was provided from all patients. We excluded patients who had any penile or pelvic surgery or radiation, coagulopathy abnormalities, uncontrolled medical or psychiatric disorders, and patients with neurological disorders. Detailed medical and psychosexual history, focused physical examination, glucose lipid profile, and morning total serum testosterone were done.

ED severity was classified based on the International Index of Erectile Function (IIEF-5) scores: 22–25, none; 17–21, mild; 8–16, moderate; and ≤ 6, severe [9]. The Erection Hardness Score (EHS) was based on self-estimated rigidity, categorized using a scale of 1–4: (1) the penis is larger but not hard, (2) the penis is hard but not hard enough for penetration, (3) the penis is hard enough for penetration but not completely hard, and (4) the penis is completely hard and fully rigid for coitus [10]. A patient-reported Self-Esteem And Relationship (SEAR) questionnaire was used to address psychosocial elements related to ED and sexual relationships [11].

Participants were randomized to the LIESWT group or Tadalafil group by the electronic method. Patients in the LIESWT group received 6 sessions (2 per week) with an average of 6,000 shocks per session (half of which were delivered to the crura penis and half to the penile shaft). This means that patients received an average of 36,000 pulses throughout treatment. Shockwaves were administered with the PiezoWave^2^ unit (Richard Wolf – ELvation Medical GmbH) with the FBL10 × 5G2 linear focusing shockwave applicator. The focus penetration depth was 15 mm. This technology accounts for the precise, well-defined focal zone. Patients were in the supine position with no anesthesia given. Patients in the Tadalafil group self-administered Tadalafil on-demand at a dose of 20mg each hour before each event of sexual intercourse. Patients in the LIESWT group were not allowed to take PDE5Is during the trial duration.

All patients were prospectively reviewed at 6 and 12 weeks. The outcomes were assessed using the IIEF-5 score, EHS, and SEAR questionnaire before and after treatment. An improvement of 5 points or greater from IIEF-5 score baseline and an increase in EHS score from 2 or less at baseline to 3 or more, and a positive change in SEAR questionnaire score were considered significant. Treatment-related side effects were also analyzed, and because we are in a developing country, the financial cost is very important, and it was also recorded.

The collected data was analyzed using the SPSS program for windows version 25 (IBM, Armonk, NY, USA). Mean, median, range, and standard deviation were used to describe continuous data, while frequency and percent were used for categorical data. Two-sample t-tests or Mann-Whitney tests and the Chi-square test were used to examine the differences between the two groups in the distributions of continuous and categorical variables. The significance level was considered at 5% (P < 0.05).

## Results

**Figure 1.**
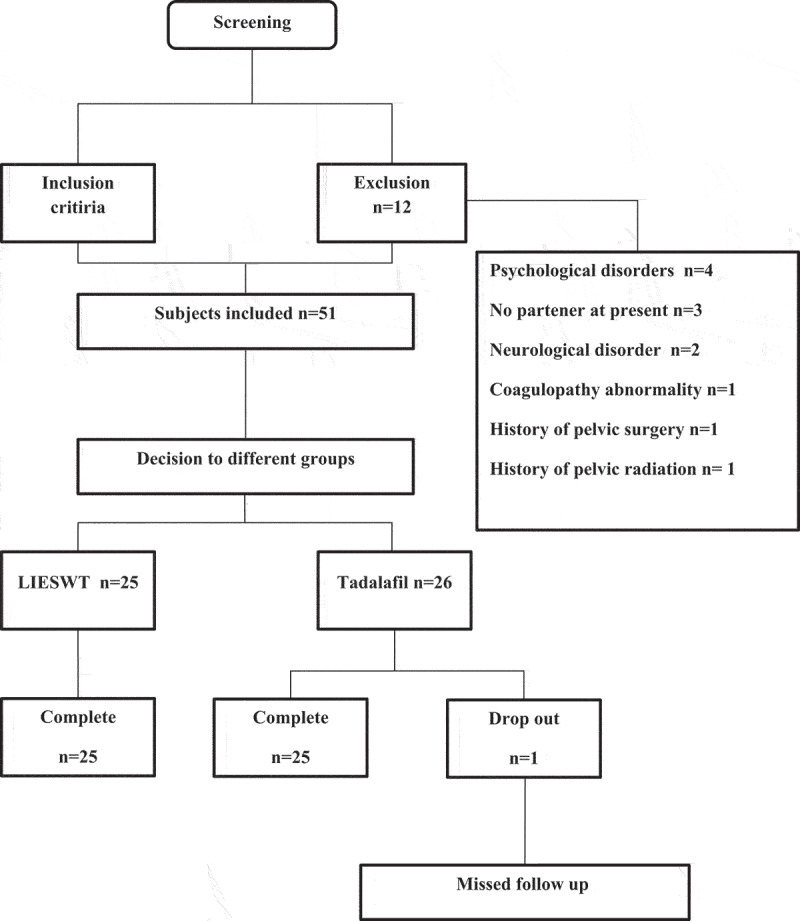
Screening, randomization and follow up flowchart.

A total of 63 participants were considered for enrollment. Fifty-one patients met our inclusion criteria, and 12 participants were excluded. A total of 25 patients were randomized into the LIESWT arm, and 26 patients were randomized into the Tadalafil arm. One patient in the Tadalafil group was dropped because of missed follow-up during the treatment period (Figure 1).

The mean age at the time of the LIESWT group was 43.7 years and in the Tadalafil group was 47 years. Participants’ baseline characteristics are presented in Table 1. There were no significant differences in baseline IIEF-5 score and baseline EHS between the two groups. The baseline IIEF-5 scores in the LIESWT and Tadalafil groups were 11.16 ± 4.2 and 10.08 ± 3.8 (p > 0.34). IIEF-5 score increased significantly from baseline to 16.36 ± 3.8 (p < 0.001) at 6 weeks and to 17.64 ± 4.00 (p < 0.001) at 12 weeks within the LIESWT group, and the IIEF-5 score also increased significantly from baseline to 17.52 ± 2.75 (p < 0.001) at 6 weeks and to 15.72 ± 3.6 (p < 0.001) at 12 weeks within the Tadalafil group. The median change in IIEF-5 score in the LIESWT and Tadalafil groups was 5.2 and 7.4 at the 6-week and 6.4 and 5.6 at the 12-week follow-up (Table 2).

**Table 1.** Participants’ baseline characteristics.

Variable	LIESWT Group	Tadalafil Group	P-value
Age (Y)	43.76 ± 10.373 (28–62)	47.08 ± 9.037 (32–63)	0.233
BMI	28.19 ± 1.43	28.66 ± 1.33	0.232
Duration of ED (Y)	5.460 ± 4.2424 (1–15)	7.600 ± 4.1231 (2–15)	0.077
Smoking	11 (44%)	13 (52%)	0.571
Diabetes	7 (28%)	11 (44%)	0.239
Hypertension	7 (28%)	4 (16%)	0.309
Cholesterol	14 (56%)	10 (40%)	0.258
Total testosterone	4.332 ± 1.5892	4.796 ± 1.9319	0.358

**Table 2.** Sexual parameters at baseline, 6 weeks and 12 weeks follow-up between LIESWT and Tadalafil on-demand groups.

Measure	Baseline		6 W follow up		12 W follow up		P value
	LIESWT Group	Tadalafil Group	LIESWT Group	Tadalafil Group	LIESWT Group	Tadalafil Group	
IIEF-5	11.16 ± 4.239 (4–18)	10.08 ± 3.81 8 (5–18)	16.36 ± 3.81 (7–22)	17.52 ± 2.75 (13–22)	17.64 ± 4.009 (8–23)	15.72 ± 3.635 (9–22)	<0.001^a,b^ 0.349^c^ 0.224^d^ 0.082^e^
EHS	1.64 ± 0.490 (1–2)	1.48 ± 0.586 (1–3)	3.1 ± 0.57 (2–4)	3.2 ± 0.46 (2–4)	3.2 ± 0.764 (2–4)	3.1 ± 0.690 (2–4)	<0.001^a,b^ 0.3^c^ 0.2^d^ 0.127^e^
SEAR	23.48 ± 2.57 (20–28)	23.84 ± 2.58 (20–28)	54.4 ± 4.25 (49–60)	57 ± 3.5 (52–63)	57.36 ± 3.79 (50–62)	54.6 ± 4.22 (48–62)	<0.001^a,b^ 0.62^c^ 0.023^d^ 0.02^e^

^a^Comparing pre and post measurement at 6 week.

^b^Comparing pre and post measurement at 12 weeks.

^c^Comparing both groups at baseline.

^d^Comparing both groups at 6 weeks follow up.

^e^Comparing both groups at 12 weeks follow up.

IIEF-5, International Index of Erectile Function-5. EHS, Erectile Hardness Score. SEAR; Self-Esteem and Relationship questionnaire.

Mean EHS also statistically higher at 6 and 12 weeks within both LIESWT and Tadalafil groups. The SEAR questionnaire scores, which show the negative effects of ED on the psychological condition and positive effects of successful treatment for both groups, were not a statistically significant difference at baseline (p = 0.3) but showed an improvement comparing baseline values to follow-up variables for both groups (P < 0.05) (Table 2).

Our patient characteristics such as age, BMI, smoking, hypertension, DM, cholesterol level, and total serum testosterone were statistically insignificant between the two groups (p > 0.05), so they cannot potentially influence the results. There was a notable statistical difference between the two groups for the safety outcomes, as the LISWT group was safe with fewer side effects than the oral Tadalafil group (p < 0.05). In the LISWT group, two patients had minimal penile pain (8%) with no need for analgesia intake, and one patient (4%) had mild penile bruises. In contrast, in the Tadalafil group, 11 (44%) of 25 patients had side effects, 5 patients (20%) suffered from muscle pain, 4 patients (16%) suffered from headache, and 2 patients (8%) suffered from nausea. For each participant, the average number of sessions in the shockwave group was 6 sessions with an average total cost of 500 U.S. Dollars, while the average of the medical treatment group was 30 tablets throughout the study costing about 62.5 U.S Dollars (p < 0.001) which is statistically significant.

## Discussion

In our study, we assessed the efficacy of LIESWT compared to oral medical treatment with Tadalafil 20 mg for our ED patients. The efficacy was comparable in both groups, with both being effective. After 6 and 12 weeks of follow-up, both groups improved by comparing baseline values to follow-up variables for all IIEF-5, EHS, and SEAR scores (P < 0.05). Tadalafil was approved in 2003 for treatment of ED and is effective after 30 minutes of administration [12]. In multiple studies, it improved erections after twelve weeks of treatment in about 81% of general ED patients taking 20 mg Tadalafil [13]. Tadalafil and other PDEI5s are the first-line treatment for male patients with ED, but they need to be taken on demand before sexual activity. Unsatisfactory results in some cases and the failure to alter the underlying ED pathology may be upsetting [2].

LIESWT provides a new non-invasive treatment pattern for ED without oral tablets taken just before sexual intercourse. Lu et al. analyzed 14 studies and showed that LIESWT significantly improved IIEF and EHS [14]. Clavijo et al. found that LIESWT also had a statistically significant improvement in IIEF score compared to participants in the sham group based on data analysis from his systematic review and meta-analysis [15]. LIESWT stimulates the release of many angiogenic factors producing regeneration of smooth muscle and endothelium of corpus cavernosum, improving penile hemodynamics, and reestablishing the patient’s trust by restoring natural erectile function [16,17]. It provides a different alternative to Tadalafil on-demand oral tablet for couples who like spontaneous rather than planned sexual activities or those who could not take oral medication because of personal non-acceptance or clinical concerns.

For the safety outcomes during the 6 and 12-week follow-up, there was a notable statistical difference between the two groups regarding the side effects as the shockwave group was with mild side effects (2 cases (8%) with tolerable penile pain, 1 case (4%) with skin bruises) and no deformity recorded, while there were noticeable side effects on the Tadalafil group (p < 0.05). In the Tadalafil group, 11 of 25 patients had side effects (44%), 5 patients suffered from muscle pain (20%), 4 patients suffered from headache (16%), and 2 patients suffered from nausea (8%). Several reviews showed that LIESWT did not experience adverse effects or discomfort [14,18]. Tadalafil’s side effects may be dose-related. Our patients had taken 20 mg Tadalafil on demand but decreased to 10 mg if side effects manifested. According to different studies and guidelines, the recommended dose is 10–20 mg and should be adjusted on-demand according to the patient’s adverse events and response [19].

For each participant, the average number of sessions in the shockwave group was 6 sessions with an average total cost of 500 U.S Dollars. In contrast, the average of the medical treatment group was 30 tablets throughout the study costing about 62.5 U.S Dollars. This cost difference is statistically significant (p < 0.001). The financial issue is vital in my country, like any developing country, because it influences patients’ decisions.

The improvements for both IIEF-5 and EHS scores revealed that both LIESWT and Tadalafil therapies successfully improved erectile function. Upgrading the SEAR score also showed that constant treatment could improve the psychological condition. According to our study analysis, LIESWT is effective, safe, and highly tolerable compared to Tadalafil. But it is more costly.

There has been no validated comparative analysis of these two treatments to our knowledge. We acknowledge the many limitations of our study, including the small number of participants. Hence, we could not analyze the results according to the degree or risk factor of ED and the short-term follow-up, so we could not judge whether the effect of LIESWT on ED was maintained after treatment.

## Conclusion

LIESWT has a comparable short-term therapeutic efficacy with higher safety outcomes than on-demand Tadalafil 20 mg for ED patients.

## Abbreviations

BMI: Body Mass Index, ED: Erectile Dysfunction, EHS: Erection Hardness Score, IIEF-5 score: International Index of Erectile Function scores, LIESWT: Low Intensity Extracorporeal Shockwave Therapy, PDE5Is: Phosphodiesterase Type 5 Inhibitors, SEAR: Self-Esteem And Relationship Questionnaire.
